# Transcriptome-Wide m6A Methylome and m6A-Modified Gene Analysis in Asthma

**DOI:** 10.3389/fcell.2022.799459

**Published:** 2022-05-30

**Authors:** Deyang Sun, Xiaolu Cai, Fenglin Shen, Liming Fan, Huan Yang, Suqun Zheng, Linshui Zhou, Ke Chen, Zhen Wang

**Affiliations:** ^1^ The First Clinical College, Zhejiang Chinese Medical University, Hangzhou, China; ^2^ Department of Respiration, The First Affiliated Hospital of Zhejiang Chinese Medical University, Hangzhou, China

**Keywords:** asthma, m6A, m6A-modified genes, MeRIP-seq, epigenetics

## Abstract

N6-methyladenosine (m6A) modification is one of the most prevalent RNA modification forms and is an important posttranscriptional mechanism for regulating genes. In previous research, we found that m6A regulator–mediated RNA methylation modification was involved in asthma; however, the specific modified genes are not clear. In this study, we systematically evaluated the transcriptome-wide m6A methylome and m6A-modified genes in asthma. Here, we performed two high-throughput sequencing methods, methylated RNA immunoprecipitation sequencing (MeRIP-seq), and RNA sequencing (RNA-seq) to identify key genes with m6A modification in asthma. Through difference analysis, we found that 416 methylation peaks were significantly upregulated and 152 methylation peaks were significantly downregulated, and it was mainly distributed in 3′ UTR. Furthermore, compared with the control group, there were 2,505 significantly upregulated genes and 4,715 significantly downregulated genes in the asthma group. Next, through a combined analysis of transcriptome and differential peaks, 14 differentially expressed genes related to RNA methylation modification were screened. Finally, through 87 health controls and 411 asthma cases from the U-BIOPRED (Unbiased Biomarkers for the Prediction of Respiratory Disease Outcomes) program, we verified three m6A-modified key genes (BCL11A, MATK, and CD300A) and found that they were mainly distributed in exons and enriched in 3' UTR. Our findings suggested that intervening in m6A-modified genes may provide a new idea for the treatment of asthma.

## Introduction

Asthma is a chronic inflammatory respiratory disease, which involves many inflammatory cells, immune cells, and cell components (Global Initiative for Asthma, 2020). It affects about 300 million people around the world and is expected to increase by one-third by 2025. It is characterized by wheezing, shortness of breath, chest tightness and/or cough, and reversible expiratory airflow restriction. These changes are usually caused by factors such as exercise, allergen or irritant exposure, weather changes, and viral respiratory infections. However, some studies have found that the incidence of asthma has certain family aggregation (Melen, 2020), and there are multiple susceptibility genes, suggesting that genetic inheritance may play an important role in the pathogenesis of asthma. Moreover, asthma has the following two characteristics in the process of onset: first, even in the same family or genetic background, the onset of its members is still random, and this randomness does not fully follow the Mendelian genetic law; and gender, heredity, and postnatal environment can affect the onset of asthma; second, whether the fetus comes on or not in adulthood is also affected by the prenatal environment, such as mother’s diet and living habits and fetal malnutrition. Several asthma susceptibility gene loci have been identified by the genome-wide association studies (GWAS). Recently (Demenais et al., 2018), an international research team including the transnational asthma genetics alliance (TAGC) found several new genomic regions with increased asthma risk. People with asthma susceptibility genes are greatly affected by environmental factors. An in-depth study of the gene–environment interaction will help to reveal the genetic mechanism of asthma.

Epigenetics plays an important role in elucidating the interaction between genes and the environment and changing the course of disease (Kabesch and Tost, 2020). It provides instructions for when, where, and how to apply genetic information (Zhang L. et al., 2020). The in-depth study on the epigenetic mechanism of asthma is conducive to investigating the relationship between genes and environmental factors and to formulating effective treatment strategies for asthma (Benincasa et al., 2021). Epigenetics mainly includes DNA methylation, RNA modification, and histone modification. N6-methyladenosine (m6A) modification is the most prevalent form of RNA modification in eukaryotic mRNA and even viral RNA (Roundtree et al., 2017; Zhao et al., 2017). m6A modification has been reported since the 1970s, but the overall distribution of the modification in RNA and its effect on gene expression regulation has been poorly understood. In 2011, the first real RNA demethylase fat mass- and obesity-associated gene (FTO) was reported, and the methylation modification of m6A was proved to be reversible, which made the study of mRNA methylation come into the eyes of scientists again (Peixoto et al., 2020). The regulatory proteins of m6A were composed of methyltransferases (writers), demethylases (erasers), and methylated reading proteins (readers) (Zaccara et al., 2019). Methyltransferases include methyltransferase-like 3 (METTL3), methyltransferase-like 4 (METTL4), and WT1-associated protein (WTAP). Their main function is to catalyze m6A modification of mRNA (Chen et al., 2019). On the contrary, the function of demethylases is to demethylate the bases that have undergone m6A modification; it includes FTO and human AlkB homolog H5 (ALKBH5) (Lan et al., 2019). Methylated reading proteins are mainly proteins of the YT521-B homology (YTH) domain family; its main function is to identify the bases that undergo m6A modification and thus activate downstream regulatory pathways such as RNA degradation and miRNA processing (Wu et al., 2019).

Abnormalities of these regulators can affect mRNA in many aspects, including structure, splicing, translation, and stability, leading to the occurrence of disease (Karthiya and Khandelia, 2020), such as pancreatic cancer (Guo et al., 2020), cervical cancer (Wang et al., 2020), and gastric cancer (Zhang B. et al., 2020). These studies suggest that the expression changes of key genes related to m6A regulators function may lead to phenotypic alteration. In addition, our previous research has shown that m6A regulator–mediated RNA methylation modification was involved in asthma (Sun et al., 2021). In this study, we will continue to investigate the relationship between m6A and asthma; we systematically evaluated the transcriptome-wide m6A methylome and m6A-modified genes in asthma by methylated RNA immunoprecipitation sequencing (MeRIP-seq) and RNA sequencing (RNA-seq).

## Methods

### Mouse Model

We modeled (Kianmeher et al., 2016; Liu et al., 2021) two groups of female 6-week-old BALB/C mice: asthma group and blank control group (*n* = 3 per group). They had the same feeding conditions and growth environment. Immunization solution: dissolve 20 mg ovalbumin (OVA) in 1 ml normal saline (NS); after OVA is completely dissolved, dilute 0.4–10 ml and mix well; and then mix it with the same volume of liquid aluminum adjuvant and place on a shaking table at 4°C for 30 min. Challenge solution: add 0.5 g OVA in 10 ml NS, fully dissolve it, and shake it on a shaking table at 4°C for 30 min. Immunization: mice were injected intraperitoneally on days 0 and 12, each with 0.2 ml, and the control group was treated with an equal volume of normal saline. Challenge: on days 18–23, the mice were atomized by ultrasound in a closed container at a dose of 10 ml once a day for 20 min. Lung tissue was taken 24 h after last atomization and immediately stored in liquid nitrogen. All experimental procedures used in this study were approved and conducted according to the Guidelines for the Care and Use of Laboratory Animal Management and Ethics Committee of Zhejiang Chinese Medical University.

### Experimental Procedure

RNA extraction and MeRIP-seq were conducted by the Beijing Genomics Institute. In brief, total RNA was extracted using the TRIzol reagent (Invitrogen), and the concentration and integrity of total RNA were measured by using the Qubit RNA HS Assay kit and an Agilent 2100 Bioanalyzer (Agilent Technology), respectively. For MeRIP experiment, about 10 ug of total RNA from each sample was fragmented using 10X RNA fragmentation buffer (Invitrogen, AM8740) by incubating in a preheated thermal cycler for 10 min at 70°C. The fragmented RNA was purified by using a RNA Clean & Concentrator™ kit (Zymo, R1018). Protein A and protein G magnetic beads (Invitrogen, 10002D, 10004D) were washed twice with IP buffer (150 mM NaCl, 10 mM Tris–HCl pH 7.5, and 0.1% IGEPAL CA-630 in nuclease-free water) before incubating with 5 ug m6A antibody (Synaptic Systems) at RT for 10 min. After two washes with IP buffer, antibody–bead complexes were resuspended in 500 μl of the IP reaction mixture including fragmented total RNA, and incubated for 4 h at 4°C. The immunoprecipitated m6A RNA with protein A/G magnetic beads was then washed three times with IP buffer for 10 min each at 4°C. Then, the beads complexes were resuspended in 500 μl reagent and collected by using the RNA Clean & Concentrator™ kit. For library preparation, the MeRIP libraries comprising eluted RNA were constructed using the SMARTer Stranded Total RNA-Seq Kit version 2 (Takara/Clontech), according to the manufacturer’s protocol. In brief, the eluted m6A RNA and input RNA were directly used for first-strand cDNA synthesis without fragmentation. After that, all the following steps were based on the manufacturer of the SMARTer Stranded Total RNA-Seq Kit version 2. Libraries for IP RNA were PCR amplified for less than 16 cycles and input libraries for less than 12 cycles. All libraries were analyzed by using an Agilent 2100 Bioanalyzer (Agilent Technologies) and quantified by using real-time PCR. Finally, the different libraries were pooled according to effective concentration and target downstream data volume and then sequenced on the HiSeq platform with the PE150 sequencing strategy.

### Bioinformatic Analysis

After obtaining the original offline data, we performed bioinformatics analysis. First, we used Trim Galore software (version: 0.5.0) to carry out quality control steps such as removing connector sequences and low-quality bases from the original data. The parameters used were: --stringency 4 --quality 22—clip R1 13 –clip R2 13 --length 30 --paired. Hisat2 software (version: 2.1.0) (Kim et al., 2015) was used for reading alignments, and the reference genome was from the UCSC RefSeq database. Second, the m6A-modified area was detected by exomePeak software (version: 2.1.2) (Meng et al., 2014), and the parameters used were window width = 200, sliding step = 30, fragment length = 150, and fold enrichment = 1.5. Third, the differential m6A modification was identified by the R package exomePeak; it combined the peaks of the samples to be compared, calculated the cumulative number of reads in the combined peak of each sample, standardized these reads, made two groups of samples at a comparable level, and then tested whether there was a significant difference in the number of reads between the two groups of samples within the peak. The parameters used were window width = 200, sliding step = 30, fragment length = 150, diff peak abs fold change = 2, fold enrichment = 1.5, and fragment length = 200; the differential m6A modification was analyzed by annotation, distribution statistics, and motif identification. Fourth, we used StringTie software (Pertea et al., 2016) to calculate the expression of mRNA and displayed it with TPM, where TPM = Ri/Li*(1e6/sum (Ri/Li)). For lncRNAs, we used previously published methods (Yang et al., 2017); in brief, transcripts were first assembled using StringTie (Pertea et al., 2016) to obtain all transcripts in each sample and then labeled. The coding ability of candidate lncRNAs was then predicted using CNCI (Sun et al., 2013) and CPC (Kong et al., 2007) software, and the expression of lncRNAs was standardized using TPM. For circRNA, we used find_circ (Memczak et al., 2013) and CIRCexplorer2 (Zhang et al., 2014) for identification and calculated its expression using SRPBM (Zheng et al., 2016), where SRPBM = Ri/(Rtotal*Li). After obtaining the gene expression of each sample, differential gene analysis was carried out by edgeR software, and then the real differential genes were screened by threshold. Finally, the overlap of differential m6A-associated genes and differentially expressed genes was analyzed.

### Validation of Clinical Significance of Gene Expression Regulated by m6A Modification

Data for validation were obtained from U-BIOPRED (Unbiased Biomarkers for the Prediction of Respiratory Disease Outcomes) (Bigler et al., 2017), a multicenter prospective cohort study involving 16 clinical centers in 11 European countries, and downloaded from Gene Expression Omnibus datasets (https://www.ncbi.nlm.nih.gov/geo/). The serial number is GSE69683, and the sample type is blood. A total of 87 healthy controls and 411 asthma cases were selected. The platform used was the GPL13158 [HT_HG-U133_Plus_PM] Affymetrix HT HG-U133 + PM Array Plate. R software and annotation packages were used to obtain gene symbols of the dataset. The differential expression of genes between asthma cases and healthy controls was analyzed by using the Wilcoxon test, and the up/down/unchanged genes were visualized using the R package “ggplot2”. The potential m6A-modified genes in patients with asthma were identified by univariate logistic regression and were cut off by p < 0.05. The least absolute shrinkage and selection operator (LASSO) Cox regression was used for feature selection and dimension reduction (McEligot et al., 2020), and the risk scores of potential asthma-related genes were calculated for verification [we used the rms package (Zhang J. A. et al., 2020) for the nomogram plotting and the nomogramFormula package (Bi et al., 2020) to calculate total points and probabilities of nomogram]. Receiver operating characteristic (ROC) curve analysis was used to evaluate distinguishing performance. The m6A methylation peak was displayed by IGV software according to the TDF file of sequencing samples, and the minimum value of data range was set to 0 to remove those non-specific peaks and low enrichment peaks (Thorvaldsdottir et al., 2013).

## Results

### Data Quality Control and Comparison

The original offline data includes the splice sequences introduced during library preparation and bases with low quality. These factors will lead to fewer reads to the genome, resulting in less information, so it needs to be filtered. We used Trim Galore to control the quality of the original offline data. Q20 and Q30 in the quality control results were calculated according to the correct rate of base recognition during sequencing and were the key indicators of base quality [Calculation formula: Qphred = −10log10P (error)]. It was found that the base ratio of Q20 in the asthma group and the control group was higher than 95%, and the proportion of Q30 bases was higher than 90%, which proves that the sequencing quality of this data is good and reliable. The results are shown in Table 1. Next, we used the software hisat2 to align the clean data to the reference genome. The results are shown in Table 2.

**TABLE 1:** Quality control and data output.

Sample ID	RawReadNum	RawBaseNum	RawQ20 (%)	RawQ30 (%)	CleanReadNum	CleanBaseNum	CleanQ20 (%)	CleanQ30 (%)	CleanRate (%)
asthma1_IP	60918430	9137764500	96.54	91.84	60789772	8480888680	97.11	92.62	92.81
asthma1_Input	61409850	9211477500	97.55	93.47	61333332	8551773485	97.90	94.03	92.84
asthma2_IP	82163936	12324590400	96.78	92.16	82003994	11428272095	97.29	92.89	92.73
asthma2_Input	106448656	15967298400	97.50	93.50	106308146	14798308864	97.88	94.12	92.68
asthma3_IP	113202638	16980395700	96.88	92.64	112997170	15732981211	97.43	93.41	92.65
asthma3_Input	112860372	16929055800	97.55	93.68	112643006	15450744535	97.98	94.39	91.27
control1_IP	57616128	8642419200	96.85	92.38	57504314	8129528526	97.38	93.11	94.07
control1_Input	70093570	10514035500	97.44	93.28	69990460	9734374827	97.80	93.84	92.58
control2_IP	70438872	10565830800	96.73	92.23	70280052	9590091040	97.37	93.16	90.77
control2_Input	66857418	10028612700	97.31	93.03	66757536	9255312164	97.70	93.66	92.29
control3_IP	114528620	17179293000	96.94	92.61	114343146	16113814104	97.42	93.30	93.80
control3_Input	54572730	8185909500	97.31	93.09	54496290	7580463300	97.72	93.73	92.60

**TABLE 2:** Summary of read mapping to the reference genome.

Sample	Mapped_reads	Map_rate (%)	Uniq_reads	Uniq_rate (%)	Prop_uniq_reads	Prop_uniq_rate (%)
asthma1_Input	43318469	70.63	40818703	66.55	39255988	64.00
asthma1_IP	51512519	84.74	49413529	81.29	46629466	76.71
asthma2_Input	83008996	78.08	77582299	72.98	74416418	70.00
asthma2_IP	70497309	85.97	67565493	82.39	64126062	78.20
asthma3_Input	78786913	69.94	74694331	66.31	71871786	63.80
asthma3_IP	96446310	85.35	92620829	81.97	87746652	77.65
control1_Input	54557143	77.95	52007719	74.31	50043490	71.50
control1_IP	49541820	86.15	47907690	83.31	45503792	79.13
control2_Input	48126518	72.09	45415983	68.03	43627542	65.35
control2_IP	58889035	83.79	56272112	80.07	53264986	75.79
control3_Input	39976898	73.36	37956646	69.65	36487532	66.95
control3_IP	97264946	85.06	93776151	82.01	88706866	77.58

### Identification and Statistics of the m6A Enrichment Area (Peak)

**FIGURE 1:**
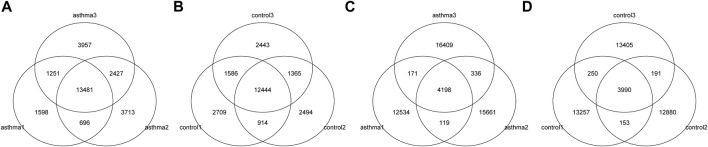
Identification and statistics of the m6A enrichment area. **(A)** Number of peaks and overlapping peaks in the asthma group. **(B)** Number of peaks and overlapping peaks in the control group. **(C)** Number of peaks and overlapping peak of modified genes in asthma groups. **(D)** Number of peaks and overlapping peak of modified genes in control groups.

MeRIP-Seq enriched and sequenced the m6A-modified region on RNA; therefore, in the m6A-modified region, the number of reads covered by IP will be significantly higher than that of the input, thus forming a “peak.” The location of m6A modification on RNA can be obtained by detecting the location of these peaks. We used exomePeak software for peak detection. The number, total length, and average length of peaks in each group were counted. The results are shown in Table 3. The overlapping of peaks between samples was analyzed (Figures 1A,B). According to the results, the number of overlapped peaks in the asthma group was 13,481 and the number of overlapped peaks in the control group was 12,444, accounting for the majority of total peaks, which proved that the consistency of m6A modification was high. At the same time, the overlap of peak modification genes in the sample was visualized (Figures 1C,D). According to the results, the number of overlapped peak modification genes in the asthma group was 4,198, and that in the control group was 3,990.

**TABLE 3:** Basic statistics of m6A enrichment peaks.

Pair	nPeak	Total length	Mean length
Asthma1	17,026	36,289,674	2,131.43
Asthma2	20,317	45,407,361	2,234.94
Asthma3	21,116	47,572,049	2,252.89
Control1	17,653	39,766,806	2,252.69
Control2	17,217	34,330,445	1,993.99
Control3	17,838	41,637,475	2,334.2
Asthma–control	568	1,302,963	2,293.95

### Identification and Analysis of the m6A Differential Peak

**FIGURE 2:**
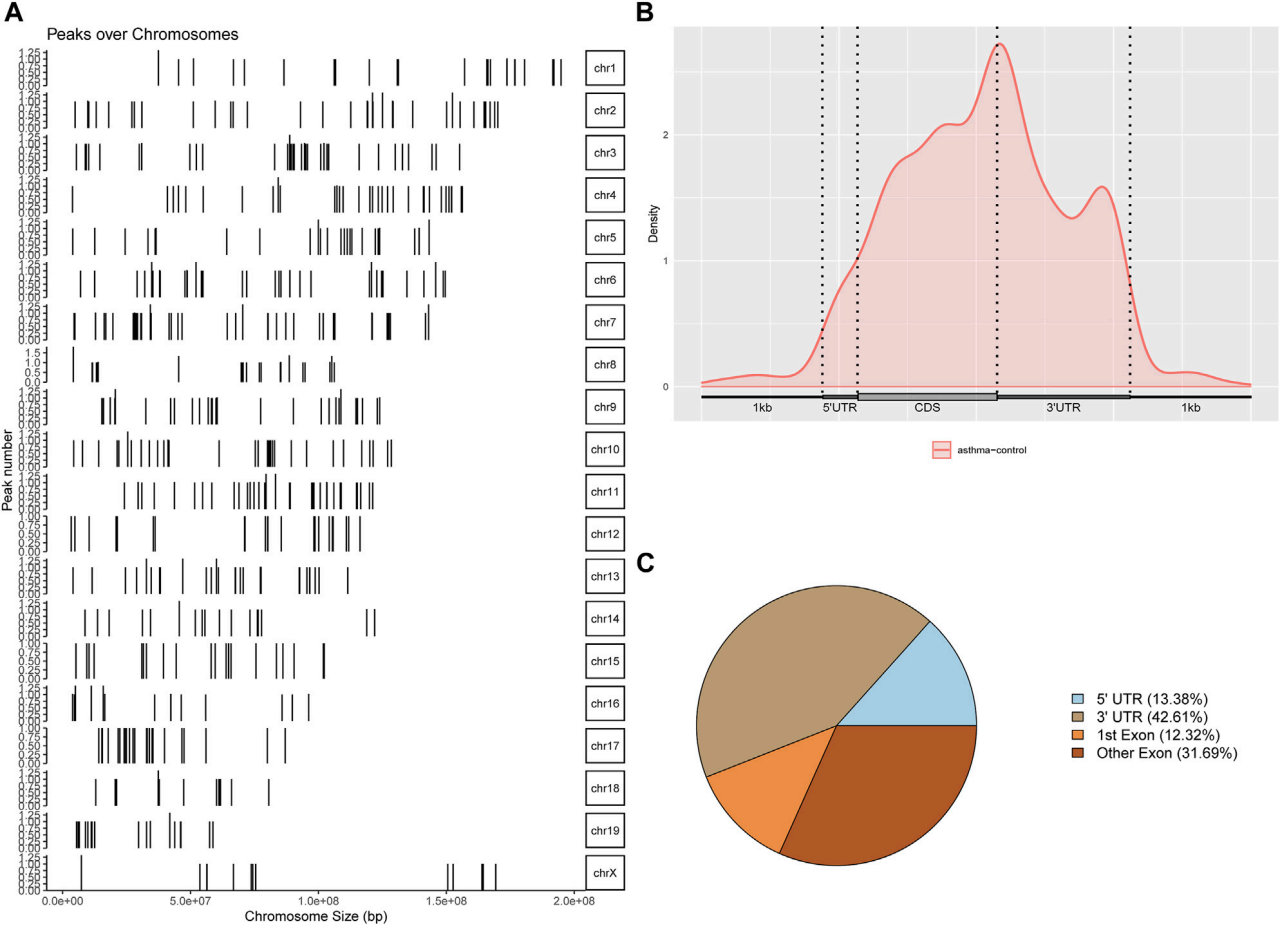
Identification and analysis of the m6A differential peak. **(A)** Distribution of differential m6A modifications on chromosomes. **(B)** Distribution patterns of differential peaks on mRNA. **(C)** Number of differential peaks distributed on each gene element.

The differential m6A modification (i.e., differential peak) was identified by using the R package exomePeak, and then the differential peak was counted by the R package Chipseeker (version: 2.16.0). A total of 568 peaks were detected, including 416 m6A methylation peaks that were significantly upregulated and 152 significantly downregulated (log2-fold change was used in this experiment to represent the ratio of standardized reads between the asthma group and the control group; the value >0 indicates that m6A modification in the asthma group is higher than that in the control group and *vice versa*). The number, total length, and average length of differential peaks between the asthma group and the control group are shown in Table 3 and Supplementary Table S1. The results of the top 20 differently expressed peaks are shown in Table 4. Next, we counted the distribution of the m6A peak. First, we counted the distribution of differential m6A modifications on chromosomes. The statistical method is to calculate the number of differential peak coverage of each base on the chromosome and draw the figure with the statistical file. (Figure 2A). Second, we analyzed the distribution patterns of differential peaks on mRNA; we found that the differential peaks were mainly distributed on CDS and 3′ UTR, and the highest peak of distribution was at the junction of CDS and 3′ UTR (Figure 2B). Finally, to understand its specific distribution on mRNA, the number of differential peaks distributed on each gene element was counted based on the location of differential m6A. This statistic helps to understand whether the distribution of differential m6A modifications has a preference for gene elements. According to the results, we found that the differential peaks had the highest percentage of 3′ UTR distribution with 42.61% (Figure 2C).

**TABLE 4:** Top 20 differentially expressed m6A peaks.

Chromosome	Peak start	Peak end	Peak region	Gene name	Fold change (log2)	Regulation	p-value
chr5	36205279	36205489	Exon	Psapl1	5.3	Up	1.30E-06
chr1	176882166	176882405	Exon	Sdccag8	4.66	Up	1.30E-08
chr11	73305470	73308725	Exon	Aspa	4.59	Up	5.20E-09
chr7	127382617	127382856	3′ UTR	9130019O22Rik	4.53	Up	3.40E-05
chr6	54269710	54269980	Exon	9130019P16Rik	4.51	Up	4.90E-05
chr2	152394932	152395053	3′ UTR	Sox12	4.36	Up	7.20E-05
chr3	100896087	100896297	3′ UTR	Vtcn1	4.3	Up	1.30E-06
chr3	88784382	88800764	Exon	5830417I10Rik	4.26	Up	5.00E-11
chr10	109824612	109852589	Exon	Nav3	4.2	Up	1.60E-06
chr2	65764571	65765081	3′ UTR	Scn2a	4.19	Up	3.20E-07
chr6	47847770	47848082	Exon	Zfp398	−2.32	Down	1.00E-11
chr5	137644169	137644589	Exon	Irs3	−2.43	Down	4.00E-17
chr6	7038873	7039263	3′ UTR	Sdhaf3	−2.58	Down	2.00E-13
chr15	32795176	32795354	Exon	Sdc2	−2.82	Down	3.50E-05
chr10	41610153	41610364	Exon	Ccdc162	−2.86	Down	3.40E-09
chr3	93396310	93396609	Exon	Rptn	−3.45	Down	1.00E-164
chr10	81391022	81391261	3′ UTR	Dohh	−3.72	Down	4.00E-11
chr15	10328323	10328894	Exon	Prlr	−3.83	Down	2.00E-07
chr13	11554091	11554332	3′ UTR	Ryr2	−3.93	Down	2.30E-06
chr14	51895156	51895277	Exon	Slc39a2	−4.04	Down	0.00016

### Identification of Motifs in the m6A Modification Region

**FIGURE 3:**
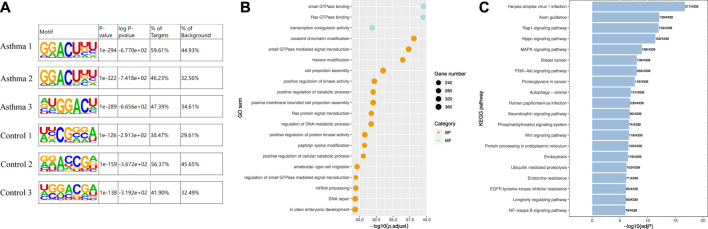
Identification of motifs in the m6A modification region and enrichment analysis of differentially m6A-modified associated genes. **(A)** Identification of motifs in the m6A modification region in all samples. **(B)** Top 20 GO enrichment analysis results of genes related to differential m6A modification. **(C)** Top 20 KEGG enrichment analysis results of genes related to differential m6A modification.

A motif refers to a short DNA sequence with a specific pattern. These sequences are likely to be important regulatory regions and related to biological functions. The m6A-modified sequence may have some characteristics. Therefore, the motif of the peak region was identified. We used motif analysis software HOMER (Hansen et al., 2016) to search motifs with high reliability in the peak area, and the width, *p*-value, and general position information of each motif in each peak sequence were obtained. The results are shown in Figure 3A. The canonical m6A motif—GGACU—was identified in the motif of the m6A-modified region in all samples of the asthma group [m6A mainly occurs on the motif of RRACH, where R = G/A; H = A/C/U (Wei et al., 2021)].

### Enrichment Analysis of Differentially Associated m6A-Modified Genes

To investigate the biological functions of differentially m6A-modified genes, we analyzed them with Gene Ontology (GO) and Kyoto Encyclopedia of Genes and Genomes (KEGG). GO is divided into three ontologies: molecular functions (MFs), cellular components (CCs), and biological processes (BPs). Figure 3B shows the results of the GO analysis, and the enrichment table is shown in Supplementary Table S2. The most significantly enriched MF was small GTPase binding, and the most significantly enriched BP was cost chromatin modification. KEGG is a simulation of the biological system, including the molecular wiring diagram of interaction, reaction, and relationship network composed of molecular structural units of genes, proteins, and compounds, as well as the information of diseases and drugs. Figure 3C shows the results of KEGG enrichment, and the enrichment table is shown in Supplementary Table S3. We found that the signal pathway with the most significant enrichment of differentially m6A-modified genes was the Rap1 (Ras-proximate-1) signaling pathway. Rap1 is a small GTPase, a small cytosolic protein that acts as a cellular switch and is essential for efficient signal transduction (Burbach et al., 2007). The latest study found that it is associated with seasonal allergies and asthma symptoms in children (Tiwari et al., 2021). In a separate study (Hu et al., 2015), asthmatic HASM (human airway smooth muscle) cells were also found to exhibit increased constitutive direct binding of the small Rap1 GTPase-activating protein Rap1GAP to the Gi protein alpha subunit, which contributes to the synergistic promotion of Ras activation. This suggests that small GTPase binding and Rap1 signaling pathway have important roles in asthma.

### Analysis of mRNA, lncRNA, and circRNA Gene Expression Levels

**FIGURE 4:**
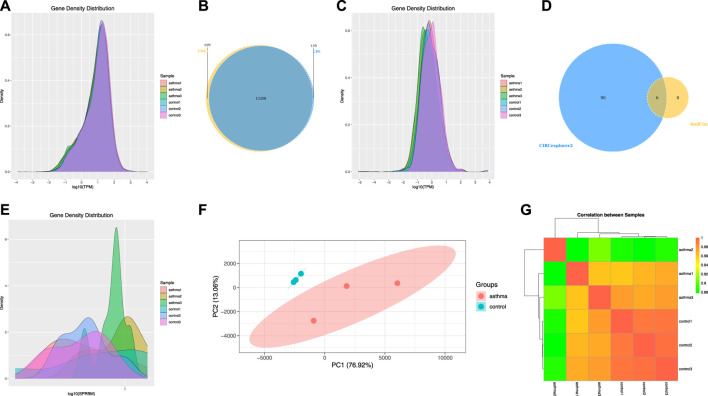
Analysis of mRNA, lncRNA, and circRNA gene expression levels. **(A)** Density map of the mRNA gene expression level. **(B)** Statistics of lncRNA transcripts. **(C)** Density map of the lncRNA gene expression level. **(D)** Statistics of circRNA transcripts. **(E)** Density map of the circRNA gene expression level. **(F)** Principal component analysis of mRNA. **(G)** Correlation between samples of mRNA.

m6A-seq input library is equivalent to RNA-seq library, which can be used to analyze gene expression and identify differentially expressed genes. Therefore, we used it to analyze the gene expression levels of mRNA, lncRNA, and circRNA. We used the software StringTie to calculate gene expression of mRNA and then standardized it with TPM and displayed it with a density map (Figure 4A). For lncRNA, we identified 13,208 lncRNA transcripts by combining the results of two prediction software (CNCI and CPC) (Figure 4B) and then standardized the expression of lncRNA with TPM and displayed it with a density map (Figure 4C). For circRNA, due to the high false-positive rate of circRNA identification, we used two software applications (find_circ and Circexplorer2) for circRNA identification, took the intersection of the results of the two software as the final prediction result, and identified a total of six circRNA transcripts (Figure 4D). Then, SRPBM was used to calculate the expression of circRNA and display it with a density map (Figure 4E). Finally, the TPM of mRNA was analyzed by a principal component analysis (PCA) (Figure 4F), and the correlation between two samples was calculated (Figure 4G). According to the results, it can be seen that the asthma group and the control group were significantly separated.

### Identification and Enrichment Analysis of Differentially Expressed Genes

**FIGURE 5:**
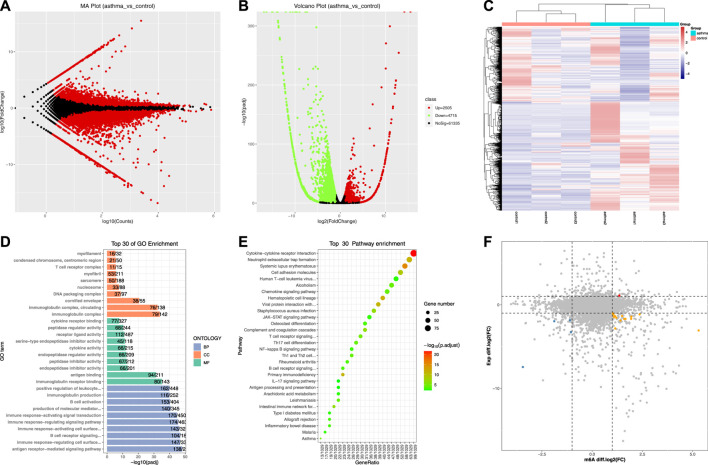
Identification and enrichment analysis of differentially expressed genes and their association with the m6A peak. **(A)** MA map of DEGs. **(B)** Volcano map of DEGs. **(C)** Heat map of DEGs. **(D)** Top 30 GO enrichment analysis results of DEGs. **(E)** Top 30 KEGG enrichment analysis results of DEGs. **(F)** Correlation analysis of differential m6A peaks and DEGs.

After obtaining the gene expression of each sample, the differential gene analysis was carried out between the asthma group and the control group. Because mRNA, lncRNA, and circRNA come from the same library, we combined these three RNAs for analysis when calculating differential expression, and there were 68,555 genes after combination. Next, the differentially expressed genes (DEGs) were identified by edgeR software [If the p-value < 0.05 and the absolute value of log2 (fold change) > 1, it is considered to be a DEG]. We found that compared with the control group, there were 2,505 significantly upregulated genes and 4,715 significantly downregulated genes in the asthma group (Supplementary Table S4). DEGs were displayed using MA map, volcano map, and heat map (Figures 5A–C). Table 5 shows the top 20 genes with the most differences in mRNA. Third, the DEGs were analyzed by GO and KEGG enrichment (Supplementary Tables S5, S6). The top 30 enrichment results are shown in Figures 5D,E. The most significantly enriched CC was an immunoglobulin complex, the most significantly enriched MF was immunoglobulin receptor binding, and the most significantly enriched BP was the antigen receptor-mediated signaling pathway. As for KEGG, the most significantly enriched item was the cytokine–cytokine receptor interaction. It is suggested that the DEGs are closely related to immune function.

**TABLE 5:** Top 20 differentially expressed genes.

Gene ID	Base	logFC	logCPM	Regulation	p-value
Clca1	5,409.2	−16.29130494	7.366387213	Up	0
Myh8	2,823.8	−15.0997089	6.172399951	Up	0
Krt4	15,711.7	−13.83891314	8.644550078	Up	0
Serpinb12	1,106.7	−13.75646784	4.828574019	Up	1.66E-309
Spink5	851.3	−13.38180603	4.453812124	Up	1.36E-280
Sprr3	829.8	−13.34531549	4.417317361	Up	1.10E-277
Dsc3	638.3	−12.97154373	4.043595817	Up	3.22E-248
Pkp1	589.7	−12.85857512	3.93067904	Up	1.65E-239
Calm4	549.5	−12.75851379	3.830703618	Up	6.70E-232
Krt5	539.5	−12.73242884	3.804642936	Up	6.48E-230
Gm2427	5.67	6.740257666	−1.896831549	Down	9.78E-10
Gm28382	5.8	6.763510877	−1.900258349	Down	9.78E-10
D830026I12Rik	5.8	6.764407151	−1.898328639	Down	9.78E-10
Gm16229	7	7.015004969	−1.710073458	Down	2.57E-11
Sgk2	7.3	7.079977219	−1.6583214	Down	4.18E-12
Gm35288	7.5	7.121622323	−1.613622066	Down	2.28E-12
Gm42417	10	7.513153512	−1.301641306	Down	3.01E-16
Gm49510	17.3	8.342220379	−0.518160378	Down	1.86E-28
Gm20507	23.7	8.799628493	−0.079795715	Down	2.43E-38
Gm28048	478	10.08616141	4.170720712	Down	3.48E-271

### Correlation Analysis of Differential m6A Peaks and DEGs

To understand the overlapping relationship between genes associated with differential m6A peak and differentially expressed genes, we carried out a series of statistics. First, the differential peaks associated with each gene (including significant and insignificant) were obtained. One gene may match with multiple peaks, and in this case, this gene will appear multiple times; there may also be no matching differential peak, in which case the gene will not appear. Second, the overlapping relationship between the genes obtained in the previous step and the differentially expressed genes was identified. We found that compared with the control group, in the asthma group, one gene with a 2-fold increase in the m6A modification level and a 2-fold increase in the expression level was shown in red, four genes with a 2-fold decrease in the m6A modification level and a 2-fold decrease in the expression level were shown in blue, and 14 genes with a 2-fold increase in the m6A modification level and a 2-fold decrease in the expression level were shown in orange. The peaks matched by these genes were all significantly different. The results are shown in Figure 5F and Table 6.

**TABLE 6:** Candidate m6A-regulated genes.

Gene	FC (differential peaks)	FDR (differential peaks)	sig	FC (differential DEGs)	FDR (differential DEGs)	Col
Bcl11a	1.15	−1.66	Yes	−2.825644379	6.44E-91	Orange
Cd300a	2.36	−2.04	Yes	−1.15051566	8.72E-15	Orange
Cdca8	1.6	−2.64	Yes	−1.687295824	4.99E-21	Orange
Gm4070	1.03	−1.86	Yes	−1.02617739	3.92E-09	Orange
Gm8989	1.08	−3.54	Yes	−1.457771396	0.001386875	Orange
Gpr183	1.2	−2.25	Yes	−1.552665067	6.39E-29	Orange
Ifi207	1.14	−2.04	Yes	−1.353868723	1.80E-20	Orange
Knl1	1.66	−1.82	Yes	−1.684556873	3.97E-29	Orange
Matk	1.41	−2	Yes	−1.88690226	4.84E-25	Orange
Ms4a6b	1.49	−3.74	Yes	−1.337626924	9.74E-24	Orange
Psapl1	5.3	−3.29	Yes	−3.069856689	5.72E-65	Orange
Serpina3f	1.04	−1.89	Yes	−1.169161036	4.82E-13	Orange
Snx20	1.06	−1.61	Yes	−1.155759676	3.84E-14	Orange
Srpk3	1.95	−1.35	Yes	−1.418677051	1.58E-20	Orange
Sh3bgr	1.35	−2.36	Yes	1.100532879	1.68E-07	Red
Gas2l3	−1.11	−1.31	Yes	−1.838909239	4.03E-30	Steel blue
Ms4a1	−1.06	−4.31	Yes	−3.283427969	3.63E-121	Steel blue
Rnf225	−1.89	−2.82	Yes	−1.968831474	9.34E-26	Steel blue
Rptn	−3.45	−1.75	Yes	−7.447536133	0	Steel blue

### Validation of Gene Expression Regulated by m6A Modification

**FIGURE 6:**
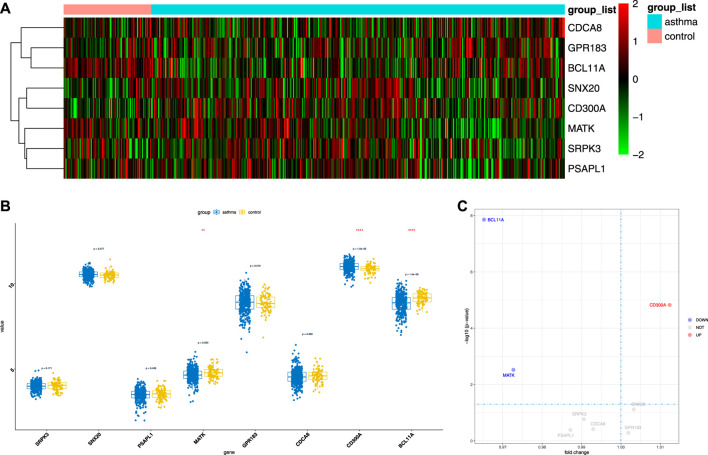
The expression of candidate m6A-regulated genes. **(A,B)** Heat map and boxplot demonstrated the expression differences of eight genes between asthma cases and healthy controls in the GEO database. **(C)** Volcano map of the expression differences of eight genes between healthy controls and asthma samples.

**FIGURE 7:**
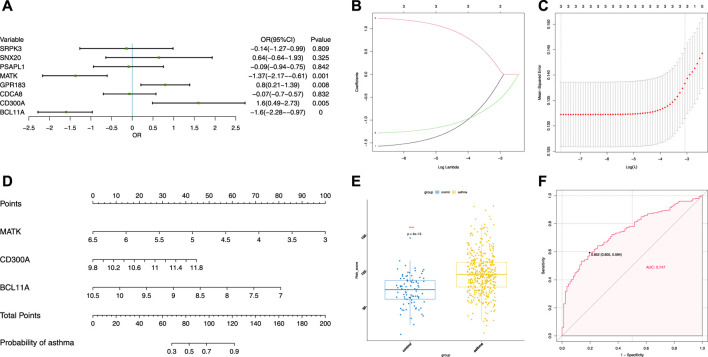
Identification of crucial genes in asthma. **(A)** Univariate logistic regression investigated the relationship between candidate m6A-regulated genes and asthma cases. **(B)** Least absolute shrinkage and selection operator (LASSO) coefficient profiles of three candidate m6A-regulated genes. **(C)** 10-fold cross-validation for tuning parameter selection in the LASSO regression. **(D)** Risk scores of three candidate m6A-regulated genes. **(E)** Risk distribution between healthy and asthma cases, in which asthma cases have a much higher risk score than healthy controls. **(F)** Discrimination ability for healthy and asthma cases by candidate m6A-regulated genes was analyzed by the ROC curve and evaluated by the AUC value.

**FIGURE 8:**
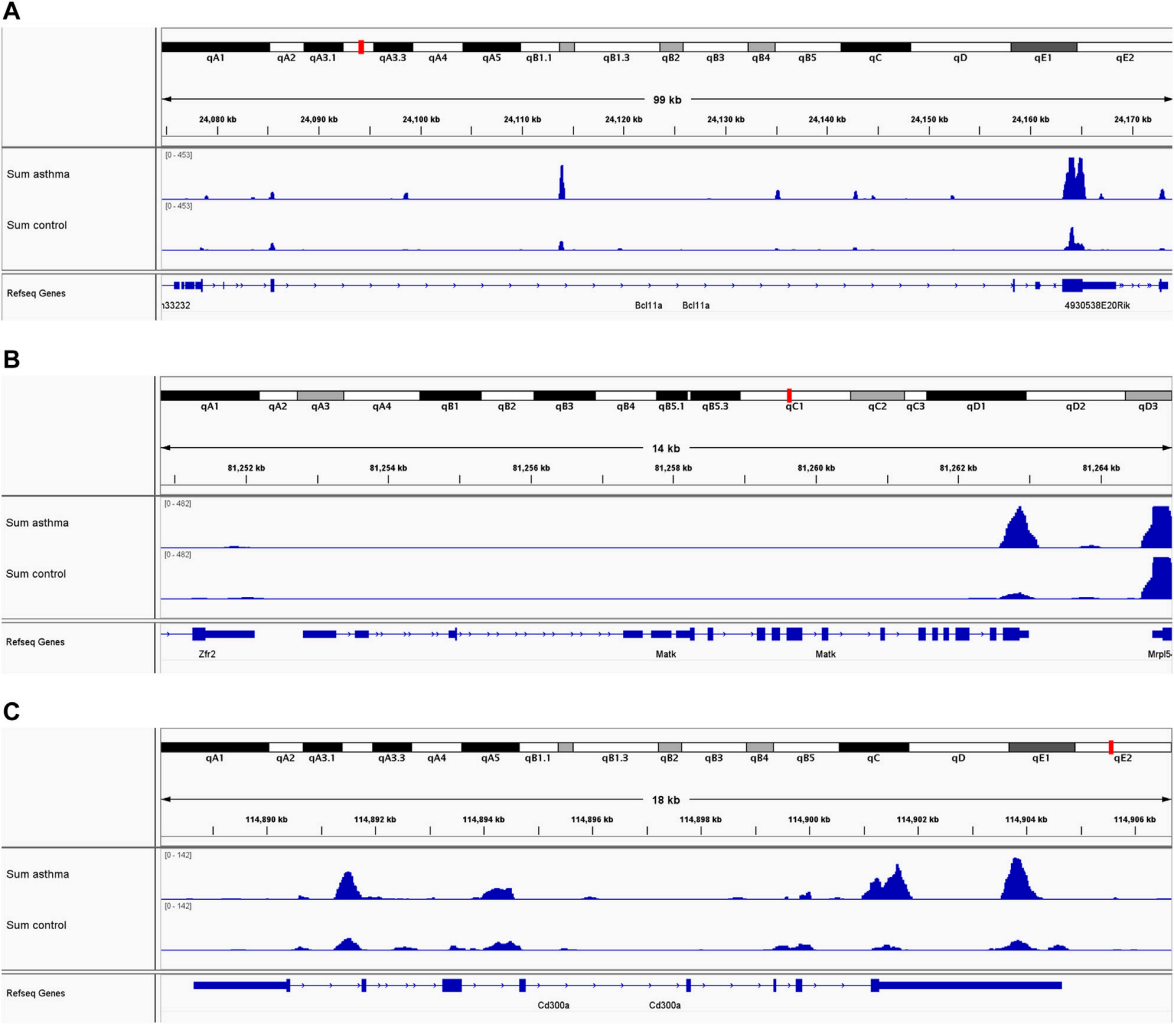
m6A methylation peaks of BCL11A, MATK, and CD300A. **(A)** Integrative genomics viewer (IGV) plots showing m6A-methylated peaks for BCL11A. **(B)** IGV plots showing m6A-methylated peaks for MATK. **(C)** IGV plots showing m6A-methylated peaks for CD300A. Blue boxes represent exons, and blue lines represent introns.

To evaluate the clinical significance of gene expression regulated by m6A modification, the GEO database was explored. We selected 14 genes with differentially methylated m6A peaks and simultaneous differential expression according to the correlation analysis of differential m6A peaks and DEGs (Wang et al., 2019; Zhang et al., 2021). Because there was very large interference inducible genes among these 14 genes, taking the intersection of MeRIP-seq and RNA-seq and GEO, it was found that there were eight co-expressed genes, of which three were differential, namely, BCL11A, MATK, and CD300A (Figures 6A–C). Next, we used univariate logistic regression to analyze these three genes and found that they were all potential m6A-regulated genes of asthma (Figure 7A). Third, LASSO Cox regression was used for feature selection and dimension reduction, and it was found that these three genes were all important for asthma (Figures 7B,C). At the same time, the risk scores of the three genes were compared between the asthma group and the healthy control group. It was found that the risk scores of the three genes in the asthma group were significantly higher than that in the healthy control group (*p* = 4e-13) (Figures 7D,E); the ROC curve also illustrated that the three genes possess a good performance in classifying patients with asthma and healthy controls (Figure 7F). Finally, the methylation peaks of BCL11A, MATK, and CD300A were visualized according to the TDF file. It was found that the methylation peaks of three key m6A-regulated genes in asthma were different from those in the control group, and they were mainly distributed in exons and enriched in 3’ UTR (Figures 8A–C).

## Discussion

Asthma is a chronic airway inflammatory disease with obvious heterogeneity and complex pathophysiological manifestations (Global Initiative for Asthma, 2020). At present, there are at least 300 million cases of asthma in the world, and the prevalence of asthma is increasing year by year. m6A RNA methylation plays an important role in regulating the expression of pathogenic genes, and abnormal m6A modification can affect RNA splicing, translocation, and translation, resulting in the occurrence of diseases. Recently (Xiong et al., 2021), the Genotype-Tissue Expression (GTEx) project reported 129 transcriptome-wide m6A profiles, covering 91 individuals and four tissues (the brain, lung, muscle, and heart). For the lung, 62 m6A quantitative trait loci (QTLs) colocalize with genome-wide association studies (GWAS) variants. Asthma-associated rs3194051 is a lung m6A QTL for immune-related interleukin-7 (IL-7) that contributes to atopic asthma, acting in bronchoalveolar lavage fluid and regulating airway eosinophilia. The results provided important insights and resources for understanding the relationship between asthma and m6A. Moreover, another study on childhood asthma found that m6A regulators also played a crucial role and screened five candidate m6A regulators (FMR1, KIAA1429, WTAP, YTHDC2, and ZC3H13) to predict the risk of childhood asthma (Xiong et al., 2021). Therefore, it is of great significance to study the relationship between asthma and m6A. In this study, a series of experiments and analyses were carried out to elucidate the transcriptome-wide m6A methylome and m6A-modified genes in asthma. This is the continuity comprehensive high-throughput transcriptome-wide analysis of m6A RNA methylation in asthma. Our study demonstrated that there were a large number of m6A modifications in asthmatic lung tissue, and further analysis showed that these modifications may play an important role in asthma by regulating gene expression.

We used the MeRIP-seq method for high-throughput sequencing of asthmatic lung tissue, and through the analysis of IP (m6A-seq library) and input (RNA-seq library), we found that there were a large number of m6A methylation peaks in the transcriptome of asthmatic lung tissue; and 568 differential peaks were detected by differential analysis, including 416 significantly upregulated and 152 significantly downregulated methylation peaks, and it was mainly distributed in 3′ UTRs. The m6A peaks were reported to be mainly concentrated in the long exons and 3′ UTRs (Dominissini et al., 2012), and our findings were consistent with this. According to the report, m6A mainly occurs on the motif of RRACH, but we found that the motif sequence of asthma was GAAUA by using Homer software, and the specific reason needs to be further studied. Next, we found that compared with the control group, there were 2,505 significantly upregulated genes and 4,715 significantly downregulated genes in the asthma group. GO and KEGG enrichment analyses showed that most of the potential functions of these genes were related to immunity, such as immunoglobulin complex and immunoglobulin receiver binding, and some pathways were known to play a vital role in asthma such as JAK-STAT (Pernis and Rothman, 2002; Chen et al., 2021), NF-κB (Edwards et al., 2009; Lertnimitphun et al., 2021), IL-17 (Silverpil and Linden, 2012; Chesne et al., 2014) signaling pathways were also enriched. Third, through the combined analysis of transcriptome and differential peak, 14 differentially expressed genes related to RNA methylation modification were screened, which were related to asthma. Finally, to evaluate the clinical significance of gene expression regulated by m6A modification, clinical samples were selected to verify the candidate gene, and it was found that there were eight co-expressed genes, of which three were differential genes, namely, *BCL11A*, *MATK*, and *CD300A*. We also used univariate logistic regression, LASSO Cox regression, risk scores, and ROC to analyze these three genes and found that they were all potential m6A-regulated genes of asthma, and the risk scores in asthma were also higher than those in healthy controls. In addition, the methylation peaks and distribution of BCL11A, MATK, and CD300A were visualized according to the TDF file, and we found that they were mainly distributed in exons and enriched in 3′ UTR. In conclusion, it was indicated that these three m6A-regulated genes play a crucial role in asthma and can affect the prognosis of asthma.

BCL11A (BAF chromatin remodeling complex subunit BCL11A) is a protein-coding gene. This gene encodes a C2H2 type zinc-finger protein by its similarity to the mouse Bcl11a/Evi9 protein. A GWAS of asthma symptoms in 1,246 children in the population of Salvador, Brazil, was carried out (Costa et al., 2015); they found that BCL11A is associated with hematopoietic symptoms of asthma, and it may be related to its interaction with BCL 6 and participation in hematopoietic cell differentiation. In addition, in another study of severe asthma (Hachim et al., 2021), they found that BCL11A was downregulated. MATK (megakaryocyte-associated tyrosine kinase) is a protein-coding gene too. The protein encoded by this gene can phosphorylate and inactivate Src family kinases and may play an inhibitory role in the control of T-cell proliferation. Esnault et al. (2013) performed a gene expression array analysis on sputum samples obtained following whole lung allergen challenge and on bronchoalveolar lavage cells obtained following segmental bronchoprovocation with an allergen found that MATK was an eosinophil-associated gene in sputum after whole lung allergen challenge. CD300A is an Ig-like receptor preferentially expressed on myeloid cells and mast cells, and it is located on chromosome 17 and contains cytoplasmic ITIMs, specifically, human and murine CD300A inhibits FcεRI-mediated signals in mast cells and basophils, resulting in the suppression of their degranulation. It was found that CD300A is a critical modulator of mast cells and eosinophil functions in allergic settings (Munitz et al., 2006). In addition, a recent study also demonstrated that CD300A-mediated signaling in iDCs was involved in Th2 responses induced by dead cells (Miki et al., 2015). In conclusion, these three genes are closely related to asthma in previous researches; in this study, we also confirmed that they are m6A-mediated key genes in asthma.

In summary, this study analyzed the transcriptome-wide m6A methylome and m6A-modified genes in asthma. It was suggested that m6A methylation may play a vital role in regulating the expression of asthma-related genes. Our research is the continuity study to comprehensively analyze the transcriptome-wide m6A methylome in asthma by MeRIP sequencing, which confirms the effect of m6A modification on asthma, opens up a new direction for using the m6A modification mechanism to study the pathogenesis of asthma, and encourages more scholars to carry out more research in this field.

## Data Availability

The datasets presented in this study can be found in online repositories. The names of the repository/repositories and accession number(s) can be found below: Bioproject ID: PRJNA778307.
